# Acoustic effects during photosynthesis of aquatic plants enable new research opportunities

**DOI:** 10.1038/srep44526

**Published:** 2017-03-14

**Authors:** Helmut G. Kratochvil, Michael Pollirer

**Affiliations:** 1 Dep. Integrative Zoologie, University of Vienna, Althanstr. 14, A-1090 Wien, Austria

## Abstract

Measurements of photosynthetic processes in hydrophytes mostly involve photosynthometers, which capture the escaping gas for subsequent analysis The most common method to detect changes in the rate of photosynthetic processes is to count the series of escaping gas bubbles. The emerging bubbles are either simply counted or they are recorded using light barriers, which is very difficult because of their small size and often varying ascent rate. The gas bubbles generated during photosynthesis by aquatic plants produce distinctive sound pulses when leaving the plants. These acoustic side effects enable completely new and highly accurate measurements. The frequency and reaction time changes of the pulses caused by external influences are therefore accurately detectable. The precise time measurements enable registering and evaluating the curves as reactions to changes in physical or chemical environmental conditions. We show that such acoustic analyses open completely new research opportunities for plant physiology.

In photosynthesis of submerse water plants Oxygen emission occurs in form of bubbles which are released from the stomata or small openings caused by injuries. Usually the oxygen discharge is in form of regular bubble series. By the observation that at the moment of escape the oxygen bubble emits a short single sound pulse the development of a new measuring method was enabled which offers completely new research options. So far the bubbles have only been counted with the stopwatch. With standard acoustic equipment for recording an analysis the time intervals from bubble to bubble can be measured accurately. Thereby the sound pulse series of extremely small bubbles can be registered. Changes in speed of oxygen emission (resp. sound pulse frequency) as consequences of changes in physical (e.g. light intensity, light quality, temperature) and chemical (e.g. environmental pollutants) conditions could be measured and registered with highest precision over a long distance and presented in form of diagrams.

Acoustic methods in plant physiology are used to study the water balance of trees^1^,^2^,^3^,^4^,^5^,^6^. This approach exploits the effect that, during changes in moisture content, the wood expands and contracts. This yields stretching sounds, which can be simply recorded with accelerometers or special microphones. Drying and water absorption in the vessels of woody plants also create cavitation noise in the ultrasonic range. These are used for studies on water balance. Similar ultrasonic noises occur during freezing and thawing of plants^2^,^7^,^8^,^9^,^10^,^11^,^12^,^13^,^14^,^15^. In marine plants, photosynthetic activity can be registered by measuring the sound attenuation caused by the rising bubbles as a whole^16^,^17^. More modern methods use, similar to medical examinations of blood vessels, the Doppler-Effect to measure the size and velocity of gas bubbles in the vessels of plants^18^,^19^,^20^,^21^,^22^,^23^,^24^,^25^,^26^.

The findings in the present study date back to observations over a period of nearly 40 years. They were made during studies of sound production in aquatic animals. Such research on underwater sounds inevitably detects noises associated with photosynthesis by aquatic plants. On sunny days in weedy areas of standing waters, other environmental sounds such as wind noise, sound signals of water insects and fish are often drowned out by the sound of gas bubbles of assimilating plants. The enormous number of oxygen bubbles creates a uniform sound image that resembles meat being cooked in a frying pan. In most macrophytes, the oxygen bubbles typically emerge in series from the stomata. The discharge frequency of pulses is very often regular, opening the possibility to determine pulse-frequencies and changes of pulse-frequencies by counting the bubbles manually or with photocells. The Canadian waterweed (*Elodea canadensis*) has been the most popular test object: parts of the plant are placed with the stem upwards in the test tube and the gas bubbles emerging from stem openings are counted.

Nonetheless, these counting methods are cumbersome, uncertain and inaccurate. Exact curves at changes in photosynthesis intensity cannot be determined because it is not possible to determine the time intervals from pulse to pulse: only the number of pulses within larger time intervals can be recorded. Our many years of work with aquatic organisms as well as through the use of video recordings showed that the typical sound pulses during photosynthesis are not caused by the passage of the bubbles through the water surface, but upon emergence from the stomata. The same effects occur when air bubbles emerge from damaged plant tissue. This provides the opportunity to establish the exact time of bubble emission.

This phenomenon proved to be the basis for exact acoustic measurements, opening entirely new possibilities for plant physiology.

## Results

**Figure 1:**
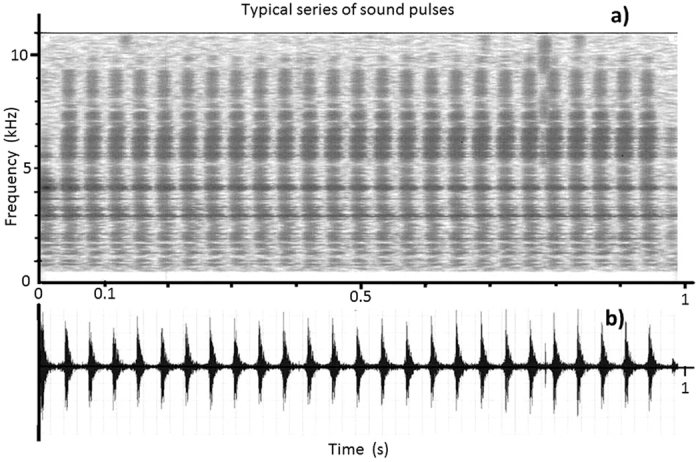
*Elodea canadensis* – Typical series of sound pulses originating from gas bubbles exiting a stoma. (**a**) sonagram, ordinate – frequency, abscissa – time. The frequency bands can be identified by the darker grays. (**b**) time-function of the same sequence, ordinate – sound pressure level, abscissa – time.

The type of bubble formation can be divided into three categories:

1. Irregular bubble emission: the time sequence of bubbles reveals no regularity. This typically involves larger-than-average bubbles.
2. Regular bubbles emission: the bubbles appear in a regular sequence (at equal intervals). This emission type may occur over a short period (a few seconds), but usually lasts much longer (up to about half an hour). In most cases the average interval time between pulses is in the range of 1–2 msec (Fig. 1).
3. Bubble emission in regular waves: the time intervals of the bubbles change periodically in the form of a pulse-frequency modulation. The periodicity of the frequency changes (the distance between the frequency maxima or the frequency minima) of the waves is equally accurate as the regular series. The temporal sequence of intervals in the modulated impulse series can either be sinusoidal or take on the form of short, regular series with intervening pauses.

### Sound parameters

Pulse length ranges from 1–3 msec, predominantly somewhat above 1 msec. The bandwidth of the time interval between pulses is very large. The smallest values may be in the range of pulse-length width when the bubbles escape from the plant in immediate succession. The largest time intervals are in the range of a few seconds. The spectrum of the very short pulses usually extends beyond the audio range (20 Hz–20 kHz), in the most pulses far into the ultrasonic range. The main energy (the place with the highest-level frequency components) of one part of the sound pulses lies in the ultrasonic range. (Figure 1) Pulse trains occasionally occur: their spectra lie exclusively in the ultrasonic range. The sound pressure levels of the regular series of pulses change only minimally. A reduced repetition rate, however, can lead to a simultaneous reduction in pulse strength. In some cases, periodic changes in level can occur at a regular pulse repetition frequency.

**Figure 2:**
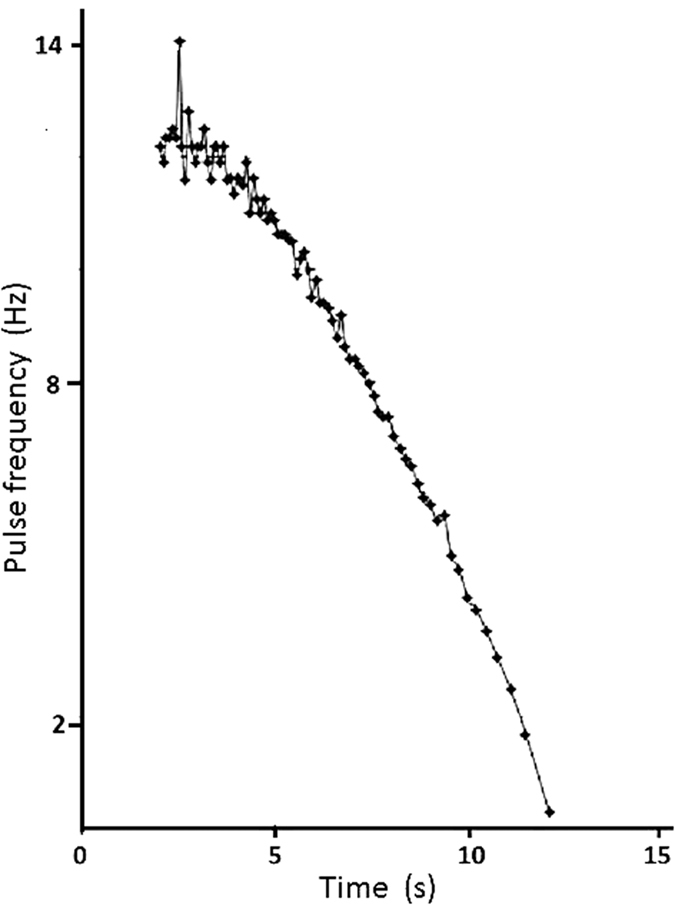
*Elodea canadensis* – Example of the applicability of acoustic methods for plant physiology. Ordinate – pulse repetition frequency, abscissa – time. The light was turned off at 0 and, after a latency period of about 2 s, the photosynthetic activity decreased, approximating a negative exponential curve.

Figure 2 shows that, after a short reaction time, the rate of bubble formation in *Elodea* after turning off the light is reduced to 0, approximating an exponential curve. The curve after turning on the light is similar. Switching the light on and off of repeats this process.

At high temperatures, other, unusual sounds occur. These apparently reflect the passage of fine gas bubbles through vessels of different cross section. They are not related to gas bubbles exiting from the stomata because they also occur if no bubble series are visible. These are usually high, harmoniously structured sounds with strong, continuous frequency changes. The physiological causes of this noise remain unknown. Possibly these are cavitation noises caused by intern gas flows.

## Discussion

**Figure 3:**
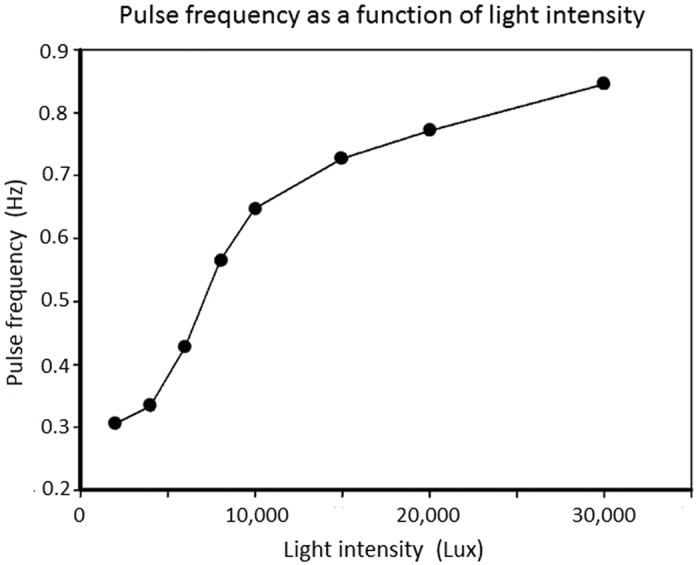
*Elodea canadensis* – Pulse rate in relation to light intensity at 28 °C. Ordinate – pulse rate, abscissa – light intensity.

The studies were mainly done with *Elodea canadensis*, which is the preferred species for experiments on photosynthesis and bubble emissions because of its robustness. Clearly, hydrophytes exhibit species-specific differences in their reactions to external conditions such as brightness, temperature and water chemistry. The mechanics of bubble discharge, however, is the same. As opposed to the cumbersome and inaccurate traditional counting methods, the acoustic method measures the time intervals very accurately. This opens up completely new possibilities for studying the metabolic processes in photosynthesis. This includes measurements of response time after switching the light on and off (Fig. 2) as a function of environmental factors such as light intensity (Fig. 3), light spectrum, temperature, nutrient levels and nutrient composition, atmospheric pressure, effect of interfering chemicals, CO_2_ content. Particularly promising is the accurate recording of pulse frequencies and their changes in response to changes of the above-mentioned factors. The curve characteristics can be compared to draw conclusions about the nature of the metabolic processes. Particularly good research opportunities arise with regard to the effects of environmental toxins on the metabolism of aquatic plants. The precise time measurements would also enable measuring the responses to low toxic dosages. Since the frequency spectrum between the pulse-series varies highly, it is possible to measure the test series when different sound-pulse trains occur in a larger plant sample. The individual impulse series can be clearly differentiated based on the intensities and frequencies spectra in the sonagrams. The sound analysis programs enable filtering out the background noise of lower quality recordings. They also allow selectively amplifying the desired sound impulse series under certain conditions. Finally, these programs can be used to cut out easily recognizable frequency components from the pulse series and to analyse the time parameters in greater detail. This provides the opportunity to develop computer programs that can measure the time parameters automatically, statistically analyse them and display them in the form of diagrams. A computer program for automatic time measurements is currently on work in cooperation with the “Institut für Schallforschung der Österreichischen Akademie der Wissenschaften”.

In any event, the method of acoustically measuring changes in oxygen delivery is to provide it with other measurement methods, e.g. Fluorometry.

## Methods

First, acoustic recordings were made in the herbaceous zone of stagnant or slow-flowing water on sunny days. Then, the exiting and rising behaviour of oxygen bubbles were studied on *Elodea canadensis* in aquariums. For comparison some other hydrophytes (*Cryptocorine, Vallisneria, Myriophyllum*) were also tested.

**Figure 4:**
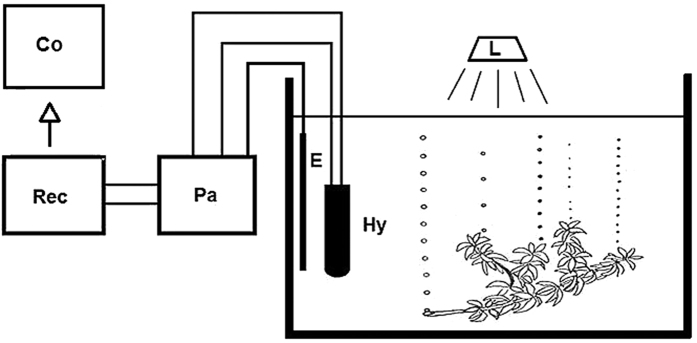
Measuring method – The sample is placed in an aquarium under controlled temperature and light conditions. The acoustic registration of the pulses does not require positioning the test plant at a certain point. When several pulse series occur, a distinction can be made based on the different acoustic frequency bands. Hy – hydrophone, Pa – preamplifier, Rec – recorder, E – electrode for the diversion of inductive interferences, Li – light.

On-and-off experiments – The light was turned on and off at regular intervals (10 min, 15 min) to detect the reaction time (time between switching on and the first sound pulse) and the time course of the “start-up” and decay of photosynthesis. The changes in the photosynthetic rate as a function of temperature and light intensity were also measured. In addition, video recordings were made, which demonstrate that the sound formation of bubbles takes place when they exit from the plant. For this purpose, small samples of *Elodea canadensis* were placed near a hydrophone in a spacious (50 L) aquarium and illuminated with full-spectrum lamps and spotlights. Thermostats and heating elements were used to change or hold constant the temperature as required. In the experiments on the temperature dependence of photosynthesis, the water temperature was continuously increased by the heat evolved by the illumination (Fig. 4).

Light intensity was kept constant or varied by changing the distance between light and plant. The hydrophone was a Brüel & Kjaer 8105 combined with a preamplifier Brüel & Kjaer 2084. The recorder was a Marantz PMD660 Professional. The sound analyses and time measurements were done using the programmes Adobe Audition 1.5, Adobe Audition 3.0 and the programme STX tools (Austrian Science System Noll u. Deutsch, Österreichische Akademie der Wissenschaften). For photometry, a lux meter Goerz MX4 was used, for temperature control a digital thermometer (HANNA HI 147–00) with an accuracy of +−0.2 °C.

## Additional Information

**How to cite this article:** Kratochvil, H. G. and Pollirer, M. Acoustic effects during photosynthesis of aquatic plants enable new research opportunities. *Sci. Rep.* **7**, 44526; doi: 10.1038/srep44526 (2017).

**Publisher's note:** Springer Nature remains neutral with regard to jurisdictional claims in published maps and institutional affiliations.
